# Casting a Wider Net: On the Utilitarian Nature of Burnout Assessment in the Workplace

**DOI:** 10.1177/01632787241259032

**Published:** 2024-05-31

**Authors:** Leon T. De Beer, Wilmar B. Schaufeli

**Affiliations:** 1 Department of Psychology, 8018 Norwegian University of Science and Technology, Trondheim, Norway; 2 WorkWell Research Unit, North-West University, Potchefstroom, South Africa; 3 Institute of Environmental Medicine, Karolinska Institutet, Stockholm, Sweden; 4 Department of Psychology, 8125 Utrecht University, Utrecht, The Netherlands; 5 Research Unit Occupational & Organizational Psychology and Professional Learning, KU Leuven, Leuven, Belgium

**Keywords:** burnout, depression, burned out, work stress, utilitarian

## Abstract

Some consider the burnout label to be controversial, even calling for the abandonment of the term in its entirety. In this communication, we argue for the pragmatic utility of the burnout paradigm from a utilitarian perspective, which advocates the greatest good for the most significant number of employees in organisations. We first distinguish between mild work-related burnout complaints and more severe burnout that can be identified in some contexts. We address the classification of burnout as an ‘occupational phenomenon’ by the World Health Organization and its ambiguous status in the ICD-11, highlighting the challenge of universally diagnosing burnout as a condition. We argue that a purely clinical approach might be too reactive as it normally only identifies employees with a diagnosable condition. We posit that early detection of burnout through valid assessment can identify struggling employees who do not yet have a diagnosable condition. This proactive approach can help prevent escalation into mental health crises and is more sensible for organisations in terms of effectiveness and employee retention.

## Commentary

For over 50 years, burnout has been described as a syndrome caused by excessive demands at work (Schaufeli et al., 2023). The word has also entered the modern zeitgeist and is widely used. According to Schaufeli et al. (2023), burnout can take two forms: ‘burnout complaints’ and ‘clinical burnout’. Burnout complaints are generally mild and primarily related to the workplace; they are rarely severe enough to prevent people from working entirely. Clinical burnout, on the other hand, is considered a more severe, medically diagnosable disorder that is frequently caused by chronic stress and may include both work-related and non-work-related factors (Van Dam, 2021). Clinical burnout can cause long-term sickness absence and necessitates personalised medical interventions, including treatment (see Schaufeli et al., 2023 for an in-depth discussion).

Unfortunately, what constitutes a universal clinical case of burnout continues to elude prescribed diagnostic manuals, notably the DSM-V (American Psychiatric Association, 2013). Nevertheless, some countries, most notably the Netherlands, have successfully incorporated criteria to identify clinical burnout cases, highly overlapping with symptoms of ‘exhaustion disorder’ (ED) in Sweden (Lindsäter et al., 2023). Exact-equivalent criteria cannot be found across all countries, calling burnout’s value as a nosological entity into question (Bianchi et al., 2023; Sen, 2022). Others have tried to present a unifying conceptualization of burnout -- as work-related exhaustion only (Guseva-Canu et al., 2021) -- but this has been criticized for lacking a proper theoretical foundation because burnout not only consists of exhaustion (inability) but also mental distance (withdrawal) (Schaufeli, 2021). In 2019, the World Health Organization (WHO) described burnout as a syndrome caused by chronic workplace stress that has not been effectively managed. Further demonstrating the ambiguous nature of burnout, the ICD-11, a disease classification list, indexes burnout as an ‘*occupational phenomenon’* rather than a medical condition.

However, when considering these challenges from a clinical standpoint, some may think of a bogus syndrome and the over-medicalization of society (e.g., Johansonn et al., 2019). Indeed, some academic circles have severely criticised burnout over the years (e.g., Bianchi & Schonfeld, 2023). From a more clinical perspective, recent research indicates that burnout may be better conceptualised as some form of depression or as job-induced depression (Bianchi & Schonfeld, 2021; Bianchi & Sowden, 2022; Martin et al., 2013). Burnout is likely to overlap with the depressive spectrum (Ahola et al., 2014), and research has shown that burnout can lead to depression over time (Shin et al., 2013). The advantage of the clinical approach is that identifying individuals in crisis follows well-established diagnostic protocols, and there are evidence-based interventions to treat various forms of depression (Bianchi et al., 2014, 2021). If the goal is only to identify employees who are currently suffering from a diagnosable mental health condition, a more clinical approach is a robust choice.

However, all this should not detract from the fact that research has shown that burnout complaints are associated with multiple undesirable physical, psychological, and occupational outcomes in many empirical research studies (e.g., Salvagioni et al., 2017). Many organisations and practitioners may view a more clinical approach as somewhat restrictive and reactive when the underlying cause is a strained relationship with work. Identifying only employees suffering from diagnosable burnout or depression does not proactively address the well-being of employees and the organisation’s problems regarding absenteeism, turnover, productivity, performance, and churn (Pirrolas & Correia, 2022). Modern organisations take a more proactive approach to well-being, attempting to identify struggling employees before they fall into the diagnosable mental health category that has implications for the individual and the organisation. This scenario underscores the wisdom of the adage, ‘prevention is better than cure’. Thus, the goal is to maximise organisational efficiency while preventing employees from reaching crisis levels by utilising early detection methods to identify when employees are struggling and what factors contribute to this.

Burnout complaints (as opposed to clinical burnout) may thus be helpful in this context because they identify employees who are at risk, allowing them to intervene and resolve their sources of distress (i.e., burnout prevention) before the employee’s capacity erodes into a mental health crisis that only becomes diagnosable later. Furthermore, the vast body of literature on burnout accumulated over the last 50 years suggests a practical application for the syndrome. As a result, we believe applying the burnout paradigm in organisations is essential from a utilitarian perspective for occupational health specialists and work-related well-being professionals in general. In this context, utilitarianism is the ethical theory that focuses on the greatest good (i.e., well-being) for the most significant number (see Scarre, 1996). This approach to workplace well-being prioritises actions and policies that aim to improve employees’ overall well-being and productivity by concentrating on early identification and referring those to employee assistance infrastructure who are already in crisis.

Whether organisations focused on the sustainability of their workforce (Harvard Business Review, 2023) will move toward a more clinical approach to managing employee well-being remains to be seen. That is, it remains an open question of the extent to which they should involve clinicians in assessing their employees’ mental health in accordance with medical standards on an ongoing basis. Notably, in the Netherlands, burnout complaints are estimated at 17% of the working population, but a diagnosis of clinical burnout is only around 2%. (Schaufeli & Verolme, 2022). As a result, our opinion is that, except where referrals are apparent, organisations should not take a more clinical approach if they want to influence the well-being of potentially struggling employees who may require intervention to prevent a downward spiral into a diagnosable mental health condition and by extension reduced organisational outcomes.

Indeed, according to a recent review by Kelloway et al. (2023), many existing mental health tools are not considered appropriate for the workplace, and employees may find such measures too invasive (Damman et al., 2015). Furthermore, formal identification and treatment of mental health conditions will most likely occur outside the workplace, and work and organisational professionals must recognise the limitations of what can be accomplished at work (Kelloway et al., 2023). Moreover, some jurisdictions do not license organisational psychology professionals to practice clinically. As a result, they may be unable to legally work with clinical depression, anxiety, or any other medically relevant diagnostic tools. Nonetheless, these professionals frequently find value in addressing burnout complaints as a non-medical entity using organisational surveys (that normally include a measure of burnout) and interventions.

However, taking such a proactive approach introduces new risks related to available resources and prevalence. One of the key criticisms levelled against burnout is the lack of universally accepted diagnostic criteria, as highlighted by Bianchi et al. (2016). This absence has led to some exaggerated estimates (for a review see: Rotenstein et al., 2018), which are clearly unrealistic and unhelpful. Burnout researchers should therefore carefully consider this issue before reporting future prevalences. We agree with the criticism that there is currently no way to universally determine the true prevalence of burnout as a condition, only an estimate of burnout complaints or risk of burnout (burnout risk). As a result, organisations must exercise caution by allocating resources to the genuinely struggling employees. However, a potential drawback of ‘casting the net wider’ with the utilitarian approach is falsely identifying too many employees as burnout risks with invalid and unreliable tools for further screening, which will exhaust or reduce the most effective use of the allocated budget. Indeed, among the identified burnout risks, some employees might recover or may later (or currently) be diagnosed with depression, anxiety, both, or another condition when their distress remains unresolved. Consequently, these employees should be referred to the appropriate employee assistance infrastructure for screening and further assistance where required. Thus, health care does not rely solely on questionnaires but also requires referral to clinical interviews and, when considered necessary, anamnesis.

Crucially, the professional’s role continues at the organisational level to identify any realistic structural changes to these employees’ work environments that can eliminate, reduce, or assist the employee in more optimally managing some of their perceived work-related stressors. Established models of employee motivation and well-being, such as the self-determination theory (SDT; Van den Broeck et al., 2021) or job demands-resources (JD-R; Bakker et al., 2023) model, can be used here. In the case of the JD-R model and the validated measures associated with it, flexible models can be estimated to determine the balance of stress in a work environment (job demands, job resources), how this affects the employee (burnout risk and work engagement), and how this eventually leads to outcomes for both the individual (well-being or distress) and the organisation (churn). This contrasts with mental health assessment tools, which typically incorporate both causes and effects into one score to offer an initial diagnosis. While not incorrect, this approach primarily focuses on diagnosing, at least preliminarily, an existing condition. However, this may not be the most effective approach for untangling subtleties within an organisation’s climate, as is often the aim of professionals in the field of work and organisational psychology.

As a result, from a utilitarian perspective, it is clear that “what ultimately matters is that people have good lives and that each person’s well-being matters equally” (Woodard, 2019, p. 211) and that implementing the burnout paradigm is therefore useful within the field of work and organisational psychology, to ensure the overall functioning of employees and modern organisations. From the utilitarian perspective, this viewpoint emphasises the value of proactive strategies for tending to employee well-being. On the other hand, a more clinical perspective may not see much value in addressing a condition labelled ‘burnout’ because the clinical perspective frequently focuses on more reactive, individualised treatment approaches, like diagnosing and treating depression. Of course, subclinical depression also exists but is usually not assessed or handled in a utilitarian way within the organisation context. Therefore, researchers, practitioners, and organisations should consider their approach carefully. All in all, both the utilitarian and clinical approaches have their respective advantages, and the decision largely depends on the professional’s and the organisation’s specific goals. However, reality is more complex than an either/or approach, and alternative perspectives on burnout may be equally, if not more, valuable when considered. Indeed, utilitarianism is not free of criticism, but there are also responses to its critics (see Woodard, 2019).

## Conclusion

In this commentary, we argue that burnout assessment is useful as a proactive strategy to optimise organisational functioning and employee work-related well-being in modern working life. Therefore, in our opinion, the responsible assessment of burnout in organisations with valid and reliable tools should continue.
